# Analysis of hindering and facilitating factors of help-seeking behavior in schizophrenia based on COM-B model: a descriptive qualitative study

**DOI:** 10.1186/s12888-023-05226-5

**Published:** 2023-10-23

**Authors:** Rui Ma, Yu Wang, Xiao-qing Wang, Kai Yu, Chen-chen Zhang, Yu-qiu Zhou

**Affiliations:** 1 Department of Nursing, Harbin Medical University, Harbin, 150081 Heilongjiang China; 2 Department of Nursing, Fu Wai Central China Cardiovascular Hospital, Zhengzhou, 451460 Henan China

**Keywords:** Schizophrenia, Help-seeking behavior, Influencing factors, COM-B, Descriptive qualitative study

## Abstract

**Background:**

Timely and systematic professional treatment is crucial in schizophrenia prognosis, but the global rate of mental health service, now, use or help-seeking behavior is low.

**Methods:**

In-depth semi-structured interviews were conducted with 13 participants with the diagnosis of schizophrenia between October to December 2021. The participants were purposively sampled from a psychiatric hospital’s. Interviews were recorded and transcribed verbatim into NVivo 12.0.

**Results:**

The findings were summarized under 3 themes and 12 subthemes: (1) capability (lack of knowledge due to insufficient mental health literacy or lack of insight, inability to access disease information due to a lack of mental health literacy, and symptoms-related barriers); (2) opportunity (lack of disease information sources, inability to balance work and study during prolonged hospitalization, accessibility and convenience of medical resources, and the acquisition and utilization of social support); and (3) motivation (awareness of the disease and professional treatment, negative experiences of disease episodes, past medical experience, confidence in tcuring the disease, and the fulfillment of daily life and self-worth).

**Conclusion:**

The medical help-seeking behavior of people with the diagnosis of schizophrenia is the result of the interaction of many barriers and facilitators, and challenges persist today. Interventions can be implemented with the BCW framework and our results to precisely eliminate delays in the diagnosis and treatment of mental problems.

## Background

The global prevalence of schizophrenia is 0.5% to 1%, the prevalence of disability is 3.75 per 1000 people, and the average life expectancy of patients is reduced by 25 years compared to the general population [1]. Furthermore, high rates of disability and hospitalization are the primary causes of poverty in patients’ families, and schizophrenia is among the top 10 diseases that cause a global burden [2]. The research findings indicated that if patients are diagnosed and treated too late, they may have more brain function impairments and poorer overall functioning and prognosis [3]. Patients often refuse to seek care after a relapse, which prolongs the disease course and leads to mental disability. Hence, receiving timely and accurate diagnosis, along with standardized treatment, is of paramount importance for their prognosis. Delays in diagnosis and treatment remain a major global problem that needs to be addressed. Addington found that the median delay in treatment for schizophrenia was 74 weeks, and 68% of people had delays of more than 6 months in the US community [4]. A survey in Qinghai province revealed that the untreated period of people ranged from 4 to 14,954 days, and the timely treatment rate of people with the onset of disease for more than 12 months was 52.66% [5]. Hansen [6] introduced a classification model for disease diagnosis and treatment delay, highlighting that factors contributing to delay primarily encompass patient-related, physician-related, and system-related factors. The existing attribution system for schizophrenia diagnosis and treatment delay often incorporates factors such as sociodemographic, healthcare resources, and biology, but its interoperability is limited [7, 8]. Moreover, it tends to overlook the subjective role people diagnosed with schizophrenia in health-promoting behaviors. Therefore, it is essential to investigate patient adversity and help-seeking behavior as vital and interminable components of diagnostic and therapeutic procedures. A uniform definition for help-seeking behavior is lacking in the academic community, and help-seeking behavior is mostly referred to in a targeted manner according to the specific research field [9]. In this study, help-seeking behavior refers to the behavior of seeking assistance from professional mental health services or psychiatrists.

Help-seeking behavior in schizophrenia can be influenced by a range of demographic characteristics, psychological, illness, and family-social factors. For example, age, economic status, marital status and literacy [10, 11]. These factors are beneficial in identifying groups that do not have easy access to mental health care, such as adolescents, people who cannot afford treatment, and unmarried people afraid of social exposure. The most common reason for incorrect attribution of illness and delayed diagnosis and treatment was insufficient knowledge or a lack of proper understanding of mental illness. If patients have high mental health literacy, psychological problems can be identified by them, making it conducive to patients seeking treatment [12]. Other variables such as attitudes and beliefs are modifiable [13]. Studies [11] have concluded that stigma and the lack of systematic and stable social support can be barriers to seeking professional help. People avoid seeking assistance due to severe stigma and fear of being labeled as “crazy,” whereas a good family support system can reduce the delay time [14]. Furthermore, a lack of mental health resources can impede patients’ access to mental health care [15].

Promoting early diagnosis and treatment for individuals with mental disorders has always been a focal point in the field of mental health. Research on the factors influencing help-seeking behavior in people diagnosed with schizophrenia has been widely applied. However, evidence indicated that independent early intervention measures, such as community interventions, multifaceted interventions, training of healthcare professionals, and interventions for clinically high-risk groups, have not significantly reduced the duration of untreated illness [16]. Therefore, further in-depth studies are still needed. A synthesis of existing studies found that they mostly lacked narrative descriptions, especially from the patient's perspective. Furthermore, the majority of these studies have used quantitative methods focused on hindering factors, with relatively few studies exploring facilitating factors [13]. Considering the perspective of people diagnosed with schizophrenia to explore the factors influencing their help-seeking behavior and uncover their real needs can enable precise and targeted interventions. Therefore, this study employs a descriptive qualitative research approach to thoroughly investigate the barriers and facilitators of help-seeking behavior in people diagnosed with schizophrenia.

The Capability–Opportunity–Motivation–Behavior model provides a comprehensive and systematic understanding of the hindering and facilitating factors in behavior [17]. It is the core of the Behavior Change Wheel (BCW), which considers three necessary conditions for behavior to occur: capability, opportunity, and motivation. Furthermore, the COM-B model revolves around nine intervention functions (including educating, persuading, training, and modeling, etc.) designed to address one or more of the issues involved as well as seven types of policies that can facilitate the implementation of these interventions. The COM-B model has been widely studied for behavior change in disease prevention, self-management, and health promotion [18, 19]. Smits [20] conducted a systematic review of the barriers and facilitators of early symptom recognition in cancer patients, mapping these factors onto the COM-B model to implement targeted interventions aimed at improving patient help-seeking behavior. The COM-B model is well-suited for the patient-centered analysis of factors influencing help-seeking behavior in this study and for proposing behavior change intervention strategies.

## Methods

### Philosophical perspective and study design

Descriptive qualitative research follows the philosophical foundations of natural inquiry, usually describing a participant’s experience directly or presenting an event in simple language [21]. When the topic under study is people’s reactions, thoughts, facilitators, or hindrances to an event, important and useful information can be obtained [22]. To understand the influencing factors of help-seeking behavior in people diagnosed with schizophrenia, it is important to explore their feelings, experiences, and perceptions related to seeking help [23]. Daily language was used to directly describe aspects of help-seeking behavioral facilitation or impairment in people diagnosed with schizophrenia to obtain more realistic raw objective results. Data analysis usually uses traditional content analysis, directed content analysis, and summary content analysis. This study used directed content analysis, a method that is both deductive and inductive; the existence of a theory or research in this approach means that it is easier to predict study variables and their relationships, as well as to aid initial coding [24]. This manuscript was prepared based on the Consolidated Criteria for Reporting Qualitative Research checklist [25].

### Participants and setting

This study involved purposive sampling from a tertiary psychiatric hospital’s outpatient and inpatient departments in mainland China between October and December 2021. In total, 13 people diagnosed with schizophrenia were enrolled. The inclusion criteria were (1) the patient received a diagnosis of schizophrenia by a psychiatrist according to the International Classification of Diseases, 10th Revision schizophrenia criteria; (2) age 18–60 years; (3) stable mental status, normal or mildly impaired self-awareness, and PANSS-G12 < 4, as judged by the attending physician; (4) ability to communicate effectively and memory normal, MMSE > 24; and (5) the participants who consented to the study. The exclusion criteria were (1) presence of severe physical illness or substance abuse and (2) comorbid with other severe mental disorders, mental retardation, or other serious organic brain diseases. Each interview lasted for 30 to 40 min, and repeat interviews were not conducted. Except for the participant and researcher, no one else was present during the interview. No participant refused to participate or dropped out from the study.

### Data gathering

Inpatient rooms and outpatient consultation rooms were selected for interviews based on participant preference.Based on a review of relevant literature, the COM-B model, and the research objectives, the research team initially formulated a pre-interview outline and conducted two rounds of pre-interviews. After the interviews, the research team consulted with relevant experts and, based on their recommendations, adjusted and revised the pre-interview outline to formulate the final interview guide for the formal interviews (Table 1). The first author collected data, audio-recorded, and took notes during the interviews. The authors had no contact with the participants before the study. The audio recordings were transcribed immediately after the interview. The transcripts were returned to the participants for comments or corrections. At the 13th interview, the point of data saturation was reached.

**Table 1:** Outline of the interview

COM-B model	Main focus
Capability	1 When you found yourself sick, what measures did you take to seek help? What was the process like?
Capability	2 What difficulties or obstacles did you encounter in seeking professional medical help? How were they resolved?
Capability	3 How do you feel about seeking professional help?
Opportunity	4 What was your family or friends' attitude toward your seeking professional help, and what were the implications?
Opportunity	5 What other external factors, aside from your own perceptions and the attitudes of those around you, do you believe would influence you to seek professional help?
Motivation	6 What are the advantages of seeking professional assistance, in your opinion?
Motivation	7 What makes you feel bad about seeking professional help?

### Data analysis

After each interview, two researchers promptly transcribed the audio recordings into textual material and imported them into NVivo 12.0. Data were analyzed using COM-B as a framework with the directed content analysis method. The themes, sub-themes, and units of meaning were all based on the COM-B model.

Steps:(1) The transcribed data were read repeatedly to familiarize the depth and breadth of the interview content; (2) The factors related to help-seeking behavior were extracted as meaningful units and codes based on the COM-B framework factor concept; (3) The initial codes were transformed into corresponding sub-themes based on similarity; (4) These sub-themes were classified under the COM-B model’s capability, opportunity, and motivation themes; (5) The extracted codes were reviewed again (merging, splitting, and deleting some sub-themes); and (6) finally, each theme was reviewed again with corresponding sub-themes and codes to ensure that no new themes were created.

### Ethical considerations

The study conformed to the ethical guidelines of the Helsinki Declaration (2013). The study received ethical approval from the Ethics Review Committee of Harbin Medical University (Institutional Review Board:HMUDQ20220517004). After explaining the study purpose, data confidentiality, and data processing to the participants, we obtained written Informed consent for voluntary participation.

### Rigour

We ensured that the study met four criteria: credibility, reliability, confirmability, and transferability. To standardize interview processes and develop researchers’ capability in qualitative analysis, the researchers attended courses and training on qualitative studies. A rapport was built with the participants during the interviews. The researcher used tools to collect data, such as audio recordings, interview transcripts, and reflective journals, to keep information safe and easily accessible for future use. After the interview, two researchers converted the interview data into text and repeatedly confirmed its accuracy. To ensure the factual value, the results of the analysis were presented to the participants for final confirmation. During data analysis, researchers made every effort to avoid bias from personal viewpoints and prior experiences, analyzing only the responses of participants. They also received supervision from the research team. For extension of the findings, we used purposive sampling to increase the diversity of participants and reported the basic characteristics of participants in detail.

## Results

### Participants

In total, 13 participants were interviewed and included in the analysis. The mean age of the participants was 40.3 years; the duration of the disease was from 1 to 30 years (median 19 years); the days in the hospital were from 6 to 130 days (mean 76 days). The demographics of the participants are presented in Table 2.

**Table 2:** Characteristics of Study Sample**(*N* = 13)**

Age, Mean (SD), range	
Years	40.3(10.5), 19–53
**Sex, N(%)**	
Female	7 (54%)
Male	6 (46%)
**Educational level, N(%)**	
University	1 (7%)
Secondary school	8 (62%)
Primary school or below	4 (31%)
**Marital status, N(%)**	
Single	10 (77%)
Married	1 (8%)
Divorced or widowed	2 (15%)
**Religion, N(%)**	
Yes	2 (15%)
No	11 (85%)
**Place of residence, N(%)**	
City	8 (62%)
Rural	5 (38%)
**Medical payment, N(%)**	
Medical insurance	7 (54%)
Publicly funded	4 (31%)
Self-funded	2 (15%)

**Fig. 1:**
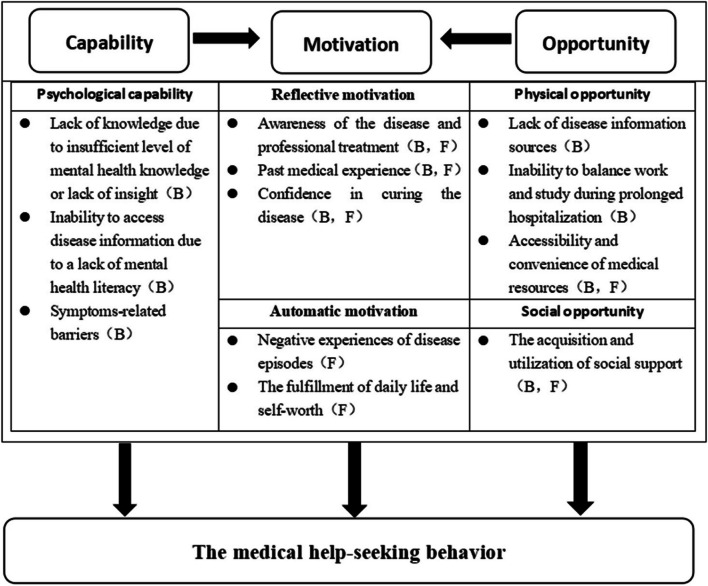
Mapping of themes to the COM-B Model. B barriers, F facilitators

The findings are summarized in three key themes of the COM-B framework (capability, opportunity, and motivation) and 12 sub-themes on barriers and facilitators of help-seeking behaviorn (Fig. 1). We found that participants lacked the capacity and opportunities related to seeking help, which hindered their help-seeking behavior. Furthermore, motivation is a prerequisite for the emergence of help-seeking behavior. The ability and opportunity factors play a crucial role in the generation and maintenance of motivational factors.

### Themes

#### Theme 1:Capability

##### Lack of knowledge due to insufficient mental health literacy or lack of insight

The public has a general lack of understanding about the prevention and treatment of mental diseases. When they experience strange symptoms, they try to rely on faith-based treatment or go to a general hospital.

“At first, I did not know where I should go. My parents took me to see a witch doctor, but it did not work. Then, we went to a traditional Chinese medicine hospital, where a doctor recommended I come here.” (N13).

Many people are hesitant to accept they are sick and refuse therapy owing to a lack of self-awareness, resulting in delays.

“I refused to go to the psychiatric hospital at first because I did not think I was sick. I was just living a stressful life, easily suspicious and mood swinging.” (N3).

##### Inability to access disease information due to a lack of mental health literacy

Because of their level of education, physical condition, and other factors, some of the participants lacked the ability to acquire and use information, and hence lacked the sense of autonomy in seeking medical care.

“I did not know where to see a doctor … I am a farmer, poorly educated, and cannot read.” (N3).

“I’m not sure whether I should come or not … My brain is not working well—unable to receive any information.” (N10).

##### Symptoms-related barriers

Mental confusion during the onset of the disease, where the patient is unable to accurately perceive their condition.

“When the disease attacked, I was in a terrible state and could not think normally.” (N1).

They may also be unable to control their behavior despite being aware of their abnormal symptoms and the need to get help.

“At that time, I began hallucinating and I considered seeing a doctor,but couldn't control myself and it didn't work out.” (N9).

However, some relapse participants had experience in this area and said that they could recognize the signs of relapse by themselves or with the help of their family members and seek professional help as early as possible.

“I told my husband if I could not sleep for a few days or you saw symptoms in me, send me to the hospital. I know I cannot think normally soon, and in 5 days I will break down.” (N3).

#### Theme 2:Opportunity

##### Lack of disease information sources

For some participants, they lack exposure to relevant illnesses, and their avenues for obtaining disease information are limited. Doubts about the accuracy of information have led participants to hesitate in seeking treatment.

“I did not know what was wrong with me; no one asked about it. Then, I discovered I was sick by checking information online, but I was not sure if it was right. I still did not know what to do.” (N2).

Even after starting treatment, there is still a lack of information on the disease.

“No one told me about this disease, but still, I talked with them [the patients] in the hospital. Then, I realized that I was really sick.” (N11).

##### Inability to balance work and study during prolonged hospitalization

Participants reported that long-term hospitalization made it difficult for them to balance work, life, and study, which reduced their willingness to seek help.

“I have many things to finish at work. However, if I stay here all the time, I will delay more things.” (N2).

“I do not want to go to the hospital because the disease recurrence delays my schoolwork. I can do many meaningful things outside, but I cannot do anything here.” (N13).

##### Accessibility and convenience of medical resources

Due to financial and geographical constraints, participants are limited to choosing nearby hospitals, and they are unable to access higher-quality mental health services.

“I had wanted my family to take me to the hospital, but I did not say anything because I knew the situation at home and could not afford to treat the disease.” (N1).

“This is the only hospital able to treat mental illness in the city. I do not know others.” (N8).

Moreover, participants’ places of residence and transportation accessibility played a role in their professional help-seeking behavior.

“My house is close to this hospital, so once I feel something is wrong with me, I will quickly come here.” (N7).

##### The acquisition and utilization of social support

Sincere support from friends and colleagues enables participants to actively cope with their illness.

“Friends and colleagues are quite supportive of me, with no strange looks, so if I relapse, I see a doctor. Better than cancer, is not it?” (N9).

The objective and subjective support from family members play a particularly important role in the patient's treatment-seeking process, bearing the significant responsibility of providing emotional comfort and tangible assistance.

“I came here mainly because of my brother. I am divorced and out of work. He provided me with financial support and encouragement over the years. Every time, it’s also he that sends me here.” (N7).

Meanwhile, if participants have inadequate social support networks that cannot provide sufficient emotional and psychological support, it can lead to various negative emotions and psychological issues.

“My father did not take me to see a doctor because he thought it could not be cured. My heart was broken. I felt very sad. My parents did not care for or love me.” (N1).

#### Theme 3:Motivation

##### Awareness of the disease and professional treatment

Participants are more inclined to seek professional help when they perceive their illness as severe and in need of treatment.

“I think the mental illness is serious and needs to be treated professionally in a psychiatric hospital.” (N5).

A correct understanding of the importance and benefits of professional treatment motivates participants to seek help.

“I think everyone probably has some mental problems. We need to see a professional doctor in time so that we can get better quickly.” (N11).

The description provided by the participant makes it evident that delays in treatment might occur when patients lack an objective, realistic grasp of their illness.

“I used to think this disease could be relieved by myself, but I did not expect to lose control and delay treatment.” (N8).

##### Negative experiences of disease episodes

Causing harm to the family makes participants feel guilty, and they seek professional help in the hope of reducing the burden on their families and gaining recognition.

“I want to heal because I broke the hearts of my family … I feel very sorry, very regretful … I also want my husband to see that I can do it.” (N7).

Social stigma can make some patients afraid to seek medical help. However, some participants also expressed that it motivates them to seek help to avoid ongoing judgment from others.

“I want to be cured completely. The mental illness does not sound very good. I do not want to live with it for the rest of my life.” (N11).

##### Past medical experience

Positive experiences from seeking help have a beneficial impact on participants, encouraging them to actively seek medical assistance when their condition relapses.

“This hospital treatment is good. I do not hallucinate now. I will see a doctor actively next time.” (N13).

However, negative experiences with the professional help-seeking process may cause some participants to no longer want to choose to seek help.

“I went to many hospitals, but there was no effect. I really do not want to see a doctor. The hospital is like a prison, making me uncomfortable and tired.’’ (N4).

##### Confidence in curing the disease

When participants believe they can overcome the illness, both their motivation and actions to seek help increase.

“I came here because I thought it could be cured. Others said it was incurable, but I think I’m past menopause and the bad days are over, so I’m sure I can.” (N12).

In contrast, some participants were skeptical of the current level of treatment and had negative feelings about professional help-seeking.

“I do not think it can be cured because there is not anyone cured around, with no exception. Why did I come here again?” (N10).

##### The fulfillment of daily life and self-worth

Most participants expressed a desire for a daily life, which was a huge motivator for them to seek professional help.

“I want to live a normal life like others, cherish life, take good care of my parents, and get along well with my siblings.” (N4).

On top of this, the desire to realize a sense of self-worth motivated participants to want to change.

“I want to start over. I think I’m a useful person. I’m still useful to the country.” (N7).

## Discussion

To gain a deeper understanding of the variables influencing the professional care-seeking behavior of people diagnosed with schizophrenia in China, this study employed a qualitative research design based on the COM-B model. As posited by the COM-B model, people diagnosed with schizophrenia can seek professional help in a timely manner when they possess both the physical and psychological capabilities and have the opportunity to access professional assistance, with motivation being a fundamental driving force in this process. Next, we will discuss the research findings in light of the framework of factors influencing help-seeking behavior, integrating intervention functions and policies.

### Competence is the prerequisite and foundation for seeking assistance

Capability includes physical and mental ability, physical ability refers to physical strength or endurance; mental ability refers to the knowledge and mental skills involved in the thinking process. In this study, we did not analyze the relationship between help-seeking behavior and physical capabilities, as older individuals or those with severe physical illnesses were excluded from the survey. Inadequate psychological skills prevented participants from generating help-seeking motivation and expressing their need for help with relevant information. Consistent with previous studies, knowledge deficits in mental health render patients vulnerable to misconceptions, such as blind superstition and reliance on lay help [10, 13]. Taking into consideration the significance of mental health literacy in professional help-seeking and its susceptibility to various influencing factors such as age, residence, and occupation [26]. We need to employ various forms of education, such as offline seminars and online media activities, to provide mental health literacy education to different target audiences [27]. Notably, there is a need to ensure the accuracy of the content of mental illness on social media to avoid people being misled by reports where rumors or bias are present [28, 29].

Furthermore, participants identified lower levels of education and cognitive impairments as reasons for their inadequate information-seeking abilities. It is widely acknowledged that education level has a significant impact on the lack of disease knowledge. It has been confirmed that schizophrenia can lead to deficits in learning, memory, and information processing speed [30]. In addition to knowledge education, incorporating medication treatment and cognitive function training is essential [31, 32]. The final indispensable aspect of help-seeking psychological skills is maintaining sound cognitive abilities, which form the foundation for motivation to seek assistance.

It was found that patients may lose their perception of reality during acute onset, which is consistent with our findings [33]. It is noteworthy that in our study, participants reached a consensus with their families that they would seek medical assistance immediately upon the observation of any signs of relapse. This underscores the critical role of collaborative monitoring by patients and their families in obtaining timely medical assistance. Patients can track their own symptoms through insight education and self-monitoring skill training [34]. Lectures and communication with other families can help their families learn to recognize and deal with symptoms [35].

### Opportunities can either facilitate or hinder the act of seeking assistance

Opportunities from external factors that motivate help-seeking behavior can be divided into the physical opportunity (time, resources, etc.) and social opportunity (words, perceptions, etc.). The opportunity factor is significant for patients because it may impair their weak help-seeking motivation. External constraints can potentially lead patients to avoid seeking medical help even when they have the willingness to do so. For instance, difficulties in accessing medical resources and information, challenges in balancing work and life, and a lack of social support can all serve as external constraints. Collaboration and support from various sectors of society is required to improve external factors. Taking into account existing research and feasibility, we would like to propose several recommendations. First and foremost, there is a need for continuous financial and healthcare support [36, 37]. There should be enhanced promotion of specialized medical services. Furthermore, the establishment of mobile healthcare service platforms, telemedicine, and other convenient forms of healthcare access should be increased. Where feasible, increasing the availability of community-level mental health services is essential. Considering the challenges in obtaining disease information and making accurate judgments, it leads to a lack of knowledge and cognitive biases among patients. Continuing to strengthen the dissemination of authoritative mental health knowledge through television media and establishing communication platforms will ensure that patients receive relevant information in a timely manner [38]. Finally, given the important role of the family in recognizing symptoms of disease and encouraging treatment [39]. Families, as part of the social support system, should be considered a crucial intervention point for reducing healthcare delays [40].

### Motivation is the factor that triggers and regulates the act of seeking assistance

Motivation is the process of brain activity that encourages and guides behavior, which is divided into reflexive motivation (planning and evaluation) and spontaneous motivation (emotions and impulses, etc.). In our study, motivational factors encompass various aspects, including awareness of the illness and treatment, negative disease experiences, help-seeking experiences, confidence in treatment, and life value needs. Among these factors, understanding the severity of the illness and the importance of professional treatment is a key determinant. Only when individuals are aware of the threat posed by mental illness and recognize the effectiveness of seeking help can they take conscious action. Consistent with the present study, it was found that patients with cognitive bias toward help-seeking usually opted for non-professional help and adversely affected their condition and subsequent treatment [41]. Furthermore, self-awareness can also influence other motivational factors. Typically, we assume that patients internalize negative societal perceptions, leading to feelings of stigma and consequently, a reluctance to seek help [33]. However, in our study, some participants, upon recognizing the value of professional treatment, transformed the low self-esteem and guilt generated by the illness into motivation to seek help. Therefore, correcting patients' negative and erroneous perceptions is of paramount importance, and this requires the assistance of professionals. Through "education," we can increase patients' knowledge of the illness and treatment and enhance their understanding [34]. Another crucial motivational factor is confidence in the cure of the illness, which can mobilize participants to seek help proactively and is associated with positive healthcare-seeking experiences. In addition, the study found that confidence in the cure of the disease enhanced the desire to return to work and society [42]. And achieving a normal life with self-worth was also a motivation for treatment for some of the participants in this study. The three motivational factors are interlocked and work together to influence help-seeking behavior. In this regard, "modeling" can be employed to share successful experiences in treating the illness, thus enhancing patients' confidence in overcoming their condition.Through "training," providing employment guidance or social interaction skills training can help individuals develop the abilities needed to reintegrate into society [43]. "Rebuilding the environment" involves symptom improvement and creating a conducive healing environment, which encourages participants to seek professional help [44].

### Strengths and limitations

To begin with, we employed qualitative research methods to comprehensively explore patients' thoughts and experiential insights into their help-seeking behavior from their perspective.

A wealth of information was obtained and provided a valuable reference for patient-centered help-seeking intervention strategies. Second, this study not only focused on help-seeking behavioral deterrents, but also explored facilitators. These factors may not necessarily be mutually exclusive, but they are all essential aspects to consider in terms of interventions for the research. Unlike previous studies, we used the COM-B model to identify factors that led people to seek help. This allowed us to make more personalized structured evidence-based behavioral interventions and other implementation strategies. However, we selected only participants who were in a mental health institution and had access to mental health care. Other implications might exist for patients who did not receive mental health care. The scope of the study population should be expanded to include culturally diverse populations, and quantitative and qualitative studies can be combined.

## Conclusion

This qualitative study based on the COM-B model explored factors influencing professional help-seeking behavior in people diagnosed with schizophrenia. The findings were summarized into three important themes (capability, opportunity, and motivation) in which people’ disease knowledge, cognition of the disease and professional treatment, and social support use played key roles and deserved attention. In this regard, this study identifies intervention functions and briefly proposes intervention strategies based on the BCW framework and research findings. Among them, coercion and restriction of help-seeking behavior change for patients with mental disorders were not elaborated on. It is suggested that trials involving multiple COM-B model components be implemented and evaluated. Furthermore, evidence of the practicality and effectiveness of intervention strategies require further evaluation.

## Data Availability

The datasets generated and analysed during the current study are not publicly available due to concerns about the privacy of people diagnosed with schizophrenia, but are available from the corresponding author on reasonable request.
